# Exploring the concurrent validity of the nationwide assessment of permanent nursing home residence in Denmark - A cross-sectional data analysis using two administrative registries

**DOI:** 10.1186/s12913-017-2535-2

**Published:** 2017-08-29

**Authors:** Anna Bebe, Anni Brit Sternhagen Nielsen, Tora Grauers Willadsen, Jens Søndergaard, Volkert Siersma, Dagný Rós Nicolaisdóttir, Jakob Kragstrup, Frans Boch Waldorff

**Affiliations:** 1 The Research Unit and Section of General Practice, Institute of Public Health, University of Copenhagen, Øster Farimagsgade 5, Post box 2099, 1014 Copenhagen K, Denmark; 2 The Research Unit and Section of General Practice, Institute of Public Health, University of southern Denmark, Odense, Denmark

**Keywords:** Nursing homes, Nursing home admittance, Nursing home entry, Nursing home referral, Nursing home placement, Validation, Validity, Denmark, Register data, Population register, Algorithm, Epidemiology

## Abstract

**Background:**

Many register studies make use of information about permanent nursing home residents. Statistics Denmark (StatD) identifies nursing home residents by two different indirect methods, one based on reports from the municipalities regarding home care in taken place in a nursing home, and the other based on an algorithm created by StatD.

The aim of the present study was to validate StatD’s nursing home register using dedicated administrative municipality records on individual nursing home residents as gold standard.

**Methods:**

In total, ten Danish municipalities were selected. Within each Danish Region, we randomly selected one municipality reporting to Stat D (Method 1) and one not reporting where instead an algorithm created by StatD was used to discover nursing home residents (Method 2). Method 1 means that municipalities reported to Stat D whether home care has taken place in a nursing home or in a private home. Method 2 is based on an algorithm created by Stat D for the municipalities where Method 1 is not applicable. Our gold standard was the information from the local administrative system in all ten selected municipalities. Each municipality provided a list with all individuals > 65 years living in a nursing home on January 1st, 2013 as well as the central personal number. This was compared to the list of individuals >65 living in nursing home facilities in the same ten municipalities on January 1st, 2013 retrieved from StatD.

**Results:**

According to the data received directly from the municipalities, which was used as our gold Standard 3821 individuals were identified as nursing home residents. The StatD register identified 6,141 individuals as residents. Additionally, 556 of the individuals identified by the municipalities were not identified in the StatD register.

Overall sensitivity for the ten municipalities in the StatD nursing home register was 0.85 (95% CI 0.84-0.87) and the PPV was 0.53 (95% CI 0.52-0.54). The municipalities for which nursing home status was based on the StatD algorithm (method 2) had a sensitivity of 0.84 (95% CI 0.82-0.86) and PPV of 0.48 (95% CI 0.46-0.50). Both slightly lower than the reporting municipalities (method 1) where the sensitivity was 0.87(95% CI 0.85-0.88) and the PPV was 0.57 (95% CI 0.56-0.59).

Additionally, the sensitivity and PPV of the Stat D register varied heavily among the ten municipalities from 0.51 (95% CI 0.43-0.59) to 0.96 (95% CI 0.95-0.98) and PPV correspondingly, from 0.14 (95% CI: 0.11-0.17) to 0.73 (95% CI 0.69-0.77).

**Conclusions:**

The overall PPV of StatD nursing home register was low and differences between municipalities existed. Even in countries with extensive nation-wide registers, validating studies should be conducted for outcomes based on these registers.

## Background

Permanent nursing home is a common term for a staffed residence for individuals who are unable to take care of themselves due to for example immobility and severe health problems [1] In a number of countries, including Denmark, permanent nursing home residents are the most frail and ill of the elderly [1–3] with a high prevalence of multimorbidity, cognitive impairment and functional limitation [3]. Hence, permanent nursing home placement may be a determining factor or an outcome in epidemiological studies [4–7]. Residency at a permanent nursing home may also be an important confounder to consider, and nursing home admission is already being used as a confounding variable in many research studies in the field of public health and medicine [5, 8]. In one study it is used to find place of death [4], in another study the association between subjective memory complaints and nursing home placement is investigated [5], additionally another paper looks at factors contributing to mortality of nursing home inhabitants [7]. Gonzales-Colaco et al were interested in the cognitive decline after nursing home admission [9]. Further, reviews have addressed nursing home residence as a relevant outcome/proxy [8, 10, 11].

Denmark is well known for their comprehensive administrative registers that can be linked to other registers and bio-banks by using a personal identification number [12]. Based on several assumptions the administratively collected data is often used to calculate other variables, which may have high face validity, but often a proper validation is lacking. In Denmark, the national authority for statistical data, Statistics Denmark (StatD), identifies individuals living in permanent nursing homes according to one of two indirect methods: 1) for municipalities reporting data about home care, persons living in nursing homes were identified by individuals who had received home care in nursing homes (and not in their own home); 2) for non-reporting municipalities, persons living in nursing homes were identified by combining the address of the individual with addresses expected to be nursing homes (based on an algorithm).

For epidemiological studies, it is important to know the validity of such indirect methods. To our knowledge, no validation studies have yet been published. Hence, we aimed to validate StatD´s register of permanent nursing home residency by using the administrative data from the municipality regarding permanent nursing home residents as a gold standard.

## Methods

### Setting

Since 1968 a unique and personal Central Personal Register (CPR) identification number has registered all persons living in Denmark in the Danish Civil Registration System (CRS) for administrative purposes. This CPR-number consists of 10 digits that include information on date of birth and sex and gives the opportunity to link various administrative registers at an individual level [12].

In Denmark, the clear majority of nursing homes are managed and owned by the municipalities and their residents are therefore billed by the municipalities. The rest of the nursing homes are managed by private organizations, but they are economically supported by the municipalities if they provide information on their residents to the municipality – if not, they do not get economic support. Since all people in Denmark have a CPR identification number and all people are registered in their municipality to be able to receive health care, pay taxes, get pension and so on, the administrative records of the municipalities can identify all individuals living in nursing homes.

#### Statistics Denmark’s identification of residency in permanent nursing home

The variable “resident of permanent nursing home” is constructed by StatD by one of two indirect methods:

#### Residents identified as persons who have received home care in a nursing home (Method 1)

At data collection, 40 of the 98 municipalities in Denmark reported data to StatD about their citizens’ use of home care and indicate where services are given. Persons who have received care in a nursing home within a specific year are labeled as nursing home residents.

#### Residents identified by living on addresses likely to be nursing homes (Method 2)

For those 58 municipalities not providing information about use of home care the identification of residents is computed from an address list of home care facilities. It is assumed that an address shared by six or more people aged 80 years or older is a nursing home, when this age group represents more than 75 % of the residents on the address, in the capital region only 50 %. StatD use three of their own registers for this algorithm: “The population register”, “Home care in home register”, and the “Home care at nursing home register”.

#### Population and data used for the validation study

Permanent nursing home residents > 65 years old were identified by their CPR-number on the 1^st^ of January 2013. We included individuals from all five regions of Denmark. Two municipalities were selected randomly from each region: one for which individuals living in nursing home facilities was based on home care data delivered by the municipality to StatD (Method 1) and one where addresses were based on StatD’s algorithm (Method 2).

**Fig. 1:**
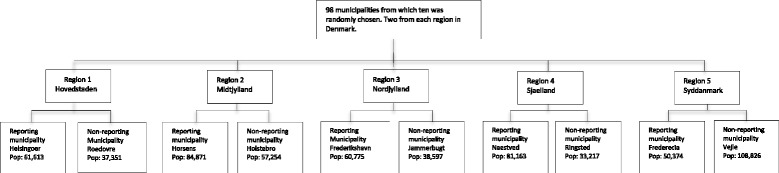
Flowchart showing the selection process of participating municipalities, which was done in a randomly selective way

The process of selecting data from the 10 municipalities within the five regions had the following steps (Fig. 1):

1. StatD was asked for data extraction including all permanent nursing home residents in Denmark on January 1st, 2013 (irrespective of the method for finding addresses of nursing homes).
2. The municipalities in each of the five regions were divided into two groups depending on the two different methods according to StatD.
3. Ten municipalities, two from each region stratified according to method for determining the addresses were randomly selected.
4. The selected municipalities where asked to provide information from the administrative systems about individuals living in nursing homes and all 10 selected municipalities agreed to participate. One municipality lacked information on the last four digits in the CPR-number for 50 persons registered as living in permanent nursing homes. However, information about names, addresses and the first six digits was sufficient information for StatD to identify the full CPR-numbers for all 50 individuals.
5. *Gold standard*

Information on permanent nursing home residents from the municipalities was used as gold standard since this information is a dedicated administrative system used to bill permanent nursing home residents for their accommodation. This register is the only register possible to use when direct knowledge about individuals in nursing homes is wanted since all individuals in Denmark are registered in their municipality to receive for example pension.

#### Statistical analysis

For the analyses, two binary variables were used:

1. Nursing home resident according to StatD. Resident of a nursing home as registered in StatD (yes/no).
2. Nursing home resident according to municipality (gold standard). Residents of a nursing home as documented by each of the 10 selected municipalities (yes/no).

First, we visualized the distribution of the population from the 10 selected municipalities and the distribution of sex, age, inhabitants >65 years old, socio-economic status, and how many inhabitants that are labeled as nursing home residents based on information from the municipality records versus the register from StatD. Information about the municipalities was obtained from StatD ´s publicly accessible tabulations (Table 1). Secondly, we examined the sensitivity and positive predictive value (PPV, with 95% confidence interval, CI) of StatD’s register, taking into consideration how the information about permanent nursing home addresses was obtained (from home care reports of the municipality or by algorithm).

**Table 1:** Population size, distribution of sex, marital status, inhabitants >65 years, and socio economic position (SEP) among participating municipalities

					*Sex*	*Age*				*Married*				
*Region*	*Municipality*	*Population* *n*	*Report*	*Stat D method used*	*Male,%*	*>65 %*	*SEP 1*	*SEP 2*	*SEP 3*	*Yes*	*SD*	*MD*	*Sensitivity*	*PPV*
Hovedstaden	Helsingør	61.613	YES	1	48.9	12.9	28.428	1.345	31.840	24.507	562	425	0.96	0.73
	Rødovre	37.351	NO	2	18.2	7.0	17.671	837	18.843	13.921	394	252	0.92	0.59
Midtjylland	Horsens	84.871	YES	1	42.7	13.9	40.324	1.908	42.639	33.270	686	508	0.87	0.65
	Holstebro	57.254	NO	2	28.5	10.2	28.129	1.021	28.104	23.580	568	158	0.51	0.14
Nordjylland	Frederikshavn	60.775	YES	1	30.4	13.9	27.557	1.389	31.829	26.138	690	468	0.73	0.50
	Jammerbugt	38.597	NO	2	19.6	7.9	17.850	934	19.813	17.134	351	276	0.83	0.65
Sjælland	Næstved	81.163	YES	1	40.0	15.3	38.065	2.009	41.089	32.443	752	522	0.93	0.65
	Ringsted	33.217	NO	2	16.5	5.4	16.124	818	16.275	13.707	153	167	0.55	0.60
Syddanmark	Fredericia	50.374	YES	1	25.1	9.5	22.503	1.445	26.426	20.015	748	352	0.82	039
	Vejle	10,8826	NO	2	54.1	18.8	52.636	2.268	53.922	44.557	1.237	693	0.95	0.53

Furthermore showing number of individuals identified as being permanent nursing home residents in the Statistics Denmark (SD) register and the Municipality Data (MD) and the sensitivity and PPV for each municipality

All statistical analyses were performed with SAS version 9.4 (*SAS Institute*, Cary, NC, USA).

## Results

Characteristics for the participating 10 municipalities are presented in Table 1.

Based on the data retrieved directly from the 10 municipalities (gold standard) a total of 3,821 individuals were identified as permanent nursing home residents while The StatD register identified 6,141 as residents. Furthermore, 556 of the individuals identified by the municipalities were not identified in the StatD register (Table 2). The overall sensitivity of StatD’s register was 0.85 (95% CI 0.84–0.87) and the overall PPV was 0.53 (95% CI 0.52–0.54) (Tables 3 and 4).

**Table 2:** The table is showing numbers of individuals identified as nursing home residents in the Municipality Data (MD), and in the Statistic Denmark’s register (SD)

		MD		
		YES	NO	TOTAL
	YES	3 265a	2 876a	6 141a
		1 971b	1467b	3438b
		1 294c	1409c	2703c
SD	NO	556a	108 158a	108 714a
		304b	61 711b	62 015b
		252	46 447c	46 699c
	TOTAL	3 821a	111 034a	115 855a
		2 275b	63 178b	65 453b
		1546c	47856c	49402c

The numbers also indicate the overlap between registered inhabitants living in permanent nursing homes in Statistics Denmark’s (SD) register and Municipality Database(MD)

Showing all ten municipalities (a), reporting municipalities (b) and non-reporting municipalities where algorithm was implemented (c)

**Table 3:** Number of individuals identified as nursing home residents by the Statistical Denmarks (SD) register and by the Municipality data (MD), and sensitivity and PPV for each municipality and in total for in non-reporting municipalities where the algorithm was implemented (Stat D´s method 2)

Municipality	Number of individuals identified as nursing home residents by SD	Numbers of individuals identified as nursing home residents by MD	Sensitivity (95% CI)	PPV ( 95% CI)
Roedovre	394	252	0.92 (0.89-0.95)	0.59 (0.54-0.64)
Holstebro	568	158	0.51 (0.44-0.59)	0.14 (0.11-0.17)
Jammerbugt	351	276	0.83 (0.78-0.87)	0.65 (0.60-0.70)
Ringsted	153	167	0.55 (0.48-0.63)	0.60 (0.53-0.68)
Vejle	1.237	693	0.95 (0.94-0.97)	0.53 (0.50-0.56)
Total	2.703	1.546	0.84 (0.82-0.86)	0.48 (0.46-0.50)

**Table 4:** Number of individuals identified as nursing home residents by the Statistical Denmarks (SD)register and by the Municipality data (MD), and sensitivity and PPV for each municipality and in total for in reporting municipalities where Stat D´s method 1 was applied

Municipality	Number of individuals identified as nursing home residents by SD	Numbers of individuals identified as nursing home residents by MD	Sensitivity (95% CI)	PPV ( 95% CI)
Helsingoer	562	425	0.96 (0.95-0.98)	0.73 (0.69-0.77)
Horsens	686	508	0.87 (0.84-0.90)	0.65 (0.61-0.68)
Frederikshavn	690	468	0.73 (0.69-0.77)	0.50 (0.46-0.53)
Naestved	752	522	0.93 (0.90-0.95)	0.65 (0.61-0.68)
Fredericia	748	352	0.82 (0.78-0.86)	0.39 (0.35-0.42)
Total	3,438	2,275	0.87 (0.85-0.88)	0.57 (0.56-0.59)

Among the municipalities, the sensitivity and PPV of the StatD register identifying permanent nursing home residents varied substantially from 0.51 (95% CI 0.43-0.59) to 0.96 (95% CI 0.95-0.98) and the corresponding PPV from 0.14 (95% CI: 0.11-0.17) to 0.73 (95% CI 0.69-0.77), respectively (Table 1).

Table 3 presents the numbers of individuals in all 10 municipalities identified as nursing home residents in the Municipality Data, and in the StatD register.

## Discussion

The validation of StatD’s permanent nursing home register showed that 85% of the individuals identified by the municipalities as residents of nursing homes (gold standard) were labeled as such by StatD. The PPV was 0.53, i.e. only 53% of those identified by StatD where registered as residents in nursing homes in the dedicated records of the municipalities. The accuracy in terms of sensitivity and PPV of StatD’s permanent nursing home algorithm was lower in the municipalities where address lists of nursing homes were created from an algorithm (method 2) as compared to municipalities who had provided information about home care for the individuals (method 1). The difference was, however, rather small. We found a high variability between the municipalities, which might be explained by the fact that municipalities have different procedures of nursing home residents, and perhaps some of the used methods have a problem in the interface to StatD.

Sensitivity, which is independent of the population studied, may be useful for validating a register but PPV is more useful on an individual level. PPV indicates how accurate the register is for a specific individual found to be a nursing home resident, and is dependent on the prevalence residency. We did not report specificity or negative predictive value (NPV) in this study since most inhabitants of a municipality do not live in a nursing home. Therefore, specificity and NPV are close to 100% and are not relevant to consider as a quality indicator of StatD’s register.

One potential limitation of our study is that our gold standard is also a “register” that has not yet been validated. However, administrative data like our municipality gold standard data are generally considered as valid data (7,8). The municipalities have a strong incentive to register all nursing home residents to collect payment from citizens and receive reimbursement from the government for individuals living in permanent nursing homes. Registers where an reimbursement is involved are often considered useful for research purposes [13]. Therefore we hypothesize that the gold standard used in this study, is trustworthy. Further, the best available administrative registers often have to be used as gold standard to validate other registers, as for example in the study by Guldberg et al, where the Danish Urogynecological database is validated, with the Danish National Patient Registry as gold standard [14].

An additional limitation could be that we only examined permanent nursing home residents on a specific day (the 1^st^ of January) and therefore the prevalence may not be representative of any other day of the year. However, the scope of our paper was not to examine the prevalence but to validate StatD’s nursing home register using dedicated administrative municipality records on individual nursing home residents as gold standard.

Danish nursing homes were established before the law of general homes for elderly was implemented, and after the year 1987, a differentiation between nursing residents and general dwellings for elderly emerged. Nursing dwellings replaced nursing homes; however, no difference exists between care-taking or nursing. Individuals are admitted to a nursing home or a nursing dwelling depending on availability [15]. In this paper both nursing homes and nursing dwellings are therefore labeled as permanent nursing homes.

Several factors may explain the misclassification found in StatD’s register. The gold standard applied in our study is based on data about residency from municipalities that are responsible for permanent nursing homes and data is used for sending bills to citizens. Such data is likely to identify residency on an exact day and can be used to calculate a prevalence of persons dwelling in nursing homes.

The StatD methods may have misclassifications for several reasons. The method based on provision of home care may include short term nursing home rehabilitation units and not solely permanent nursing home residents. Furthermore, a misclassification of place for provision of care has few direct consequences for municipalities and citizens and may therefore not be corrected. The method based on combination of addresses can give misclassification of residency due to possible errors in one or both addresses. The list of nursing home addresses based on the StatD algorithm may provide some misclassification in situations where the elderly keep their original home address when moving into a nursing home facility [16].

We have found no other studies that have tried to validate nursing home residence status data that is based on addresses. In a study validating The Danish National Patient Registry, the register was found to be a valuable tool for epidemiological research, but not without considering strengths and limitations [17]. The same national register was validated by Mason et al. They found a low completeness, which without precaution, could lead to bias [18]. Our results are in line with these other studies, since we found a rather low sensitivity and PPV. Other national Danish registers have been validated as well, as for example Uggerby et al, investigating the validity of Schizophrenia diagnosis in the Danish Psychiatric Central Research Register, which they found to be well-suited for research [19]. Lykke Petri et al validated specific data in the Danish Gynecological Cancer Database and found it sufficient for quality monitoring [20]. Another study validated variables in the National Clinical Thyroid Cancer Database, also finding it reliable to use for research at a national level [21].

Denmark has some of the most comprehensive registers in the world, and many are hosted and maintained by StatD. However, changes in the organization and provision of health services can be factors that affect some registers and their completeness. Furthermore, different definitions of variables are used by different registers indicating that the ability to make cross-overs between registers is very important. Moreover, changing of the codes used to register and the coding practices may also have an effect on the validity of the StatD’s permanent nursing home register [22, 23].

Permanent nursing home residence is already used as a confounding variable in many research studies [5, 8, 9]. In observational studies, administrative data can be used as a confounding variable or proxy for frailty which is difficult to measure in other registers [24]. Previous studies using this algorithm to identify permanent nursing home residents might have made an overestimation, meaning that the impact of what they examined had less impact than reported. For example, if one wanted to investigate if a specific diagnosis led to permanent nursing home residency, an over- estimation of the truth might have been made. Further, if there are subgroups in the data used in a study, where the accuracy between the groups are different (like the variability between municipalities in our study), the estimates of effect when nursing home placement is used as an outcome could be biased. Therefore, it would be necessary to adjust according to municipality. Additionally, regional difference may be biased. Consequently, a valid algorithm for nursing home status is of importance in epidemiologic surveys.

Implications for research: Due to our results with a low PPV, we can conclude that validation studies are important for the accuracy in studies involving register even in countries like Denmark having comprehensive registers.

As implications for future use we suggest that one needs to be careful in the interpretation of StatD´s nursing home variable, especially since it´s accuracy varies tremendously between the different municipalities.

The validity of the variable can be improved by a direct use of the municipalities´ registers by StatD,

## Conclusion

The overall predictive value of StatD’s permanent nursing home register was found to be low. When used for epidemiological studies, an overestimation of nursing home residency is to be expected. At present direct municipality-administrative registers regarding permanent nursing home residents could be more suitable for epidemiological studies.

## Abbreviations

CI: Confidence Interval
NPV: Negative Predictive Value
PPV: Predictive Value
Stat D: Statistics Denmark
